# Dirty mice better recapitulate key features of mRNA vaccine immunogenicity observed in humans

**DOI:** 10.1128/mbio.00983-26

**Published:** 2026-07-06

**Authors:** Beatriz Praena, Frances K. Shepherd, Cera A. McDonald, Devyani Joshi, Sneh Lata Gupta, Madison L. Ellis, Lilin Lai, Alberto Moreno, Shanley N. Roach, Mark J. Pierson, Adam J. Gilbertsen, Carl Davis, Mehul S. Suthar, Jens Wrammert, Ryan A. Langlois

**Affiliations:** 1 Department of Microbiology and Immunology, University of Minnesota, Minneapolis, Minnesota, USA; 2 Institute on Infectious Diseases, University of Minnesota, Minneapolis, Minnesota, USA; 3 Center for Childhood Infections and Vaccines, Children's Healthcare of Atlanta, Division of Infectious Diseases, Department of Pediatrics, Emory University School of Medicine, Atlanta, Georgia, USA; 4 Emory Vaccine Center, Emory University, Atlanta, Georgia, USA; 5 Emory National Primate Research Center, Atlanta, Georgia, USA; 6 Division of Infectious Diseases, Department of Medicine, Emory University School of Medicine, Atlanta, Georgia, USA; 7 Department of Lab Medicine and Pathology, University of Minnesota, Minneapolis, Minnesota, USA; 8 Department of Microbiology and Immunology, Emory University, Atlanta, Georgia, USA; Washington University in St. Louis, St. Louis, Missouri, USA

**Keywords:** mRNA vaccine, dirty mice, SARS-CoV-2

## Abstract

Although specific-pathogen-free (SPF) mice have traditionally been used to test candidate vaccines, recent work has demonstrated that “dirty” mice with broad microbial exposure more appropriately recapitulate human immune responses. Using a dirty mouse model in which lab mice are co-housed with pet store mice, we modeled SARS-CoV-2 mRNA vaccine responses in dirty and SPF mice. In this study, dirty mice showed reduced serum spike-binding antibody titers after prime vaccination and required a second booster dose to reach SPF-level spike antibody titers. Additionally, spike antibody titers waned faster in dirty mice in the 5 months following prime vaccination, while the neutralizing activity of these antibodies was reduced against Omicron variants, an effect that has also been observed in vaccinated humans. We further investigated the seasonality and consistency of pathogens in co-housed dirty mice, as well as the impact of serial microbial exposure on our animal model system. We found that pathogen exposure and T cell activation remained consistent over time and that a single co-housing event was sufficient to provide broad microbial exposure. This work demonstrates that the dirty mouse co-housing system is a promising, translationally representative approach to screen candidate mRNA vaccines for efficacy and durability prior to human clinical trials.

**IMPORTANCE:**

The development of mRNA vaccines during the COVID-19 pandemic dramatically reduced hospitalization and death rates for infected individuals. However, booster vaccinations were required to achieve high antibody titers, and protection waned over time. Our research leveraged a "dirty" mouse model to test whether SARS-CoV-2 mRNA vaccinations in animals with previous microbial exposure better modeled human immune responses. We found that, unlike standard SPF mice, dirty mice also require a booster vaccination to reach maximum antibody titers and experience waning serum antibody titers over time. We propose this platform as a future model for robust preclinical mRNA vaccine testing to improve immunogenicity and durability.

## OBSERVATION

Preclinical vaccination studies typically use specific-pathogen-free (SPF) animals in barrier facilities to minimize variables and improve replicability. However, “dirty” animals with broad microbial exposure better recapitulate adult human immune activity and vaccine responses (1–5). Multiple systems (6) provide microbial exposure to laboratory mice, including co-housing with pet store mice (1), “rewilding” in outdoor-mimicking environments (7), “feralizing” via outdoor environments with wild-caught mice (8), or using laboratory mice born to wild mice (“wildlings”) that maintain natural microflora (4). Vaccination studies performed in SPF and dirty mice reliably show that microbial exposure affects humoral immunity (2, 3, 5), aligning with human data where diverse geographic and microbial exposures alter vaccine outcomes (9, 10). As humans naturally experience diverse microbial interactions, vaccination research should consider pre-existing immune responses to improve the translational potential.

**Fig 1:**
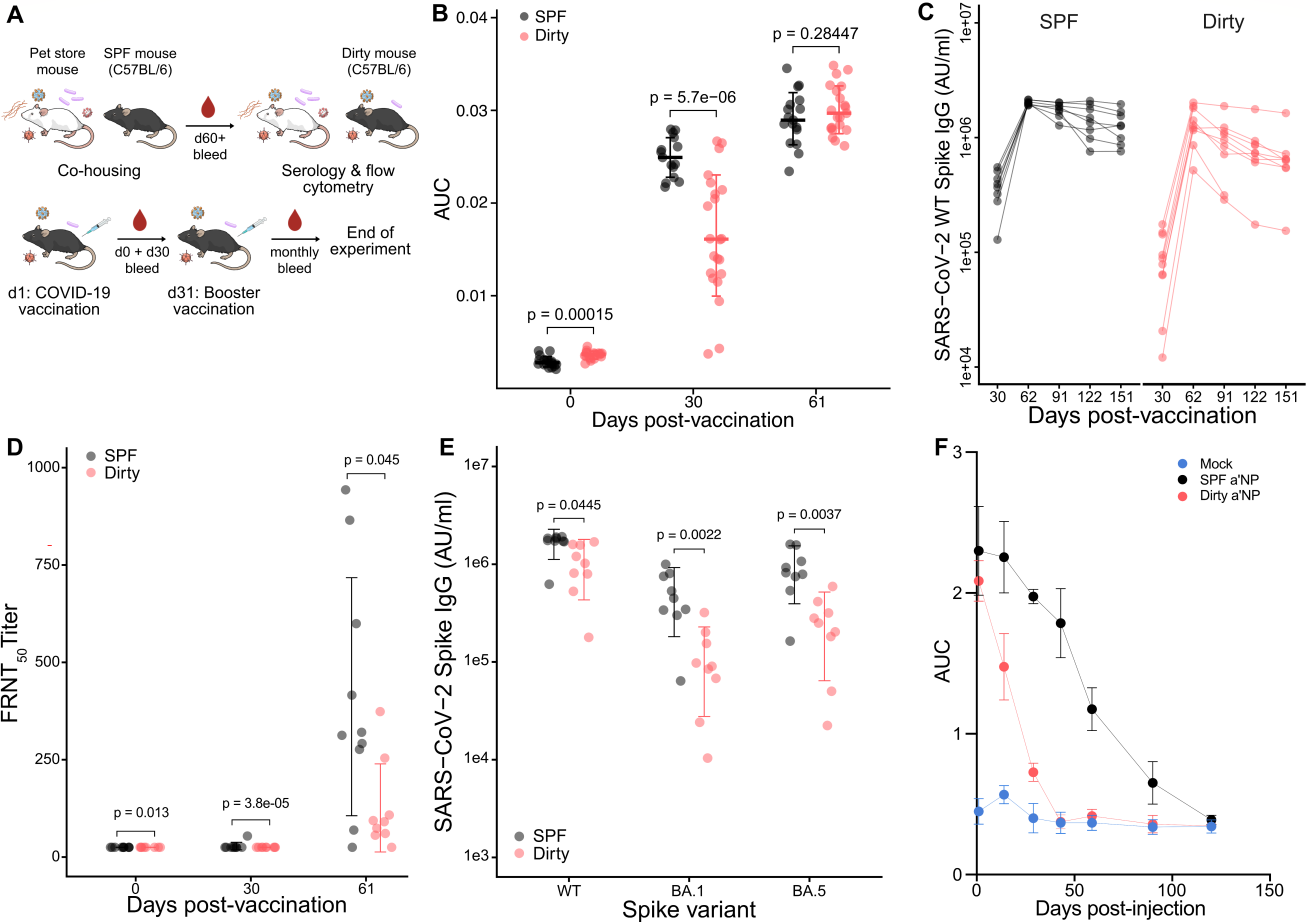
mRNA vaccination in dirty mice requires booster vaccination and is less durable than in SPF mice. (**A**) Model for generating and vaccinating dirty mice, days (**D**) indicated. (B–E) Serum antibody responses in SPF (black) and dirty (red) mice. (**B**) Anti-S1 RBD IgG levels 30 days post-prime and 30 days post-boost (day 61, *N* = 48); area under the curve (AUC) from serial dilution ELISAs (OD_405_). (**C**) Quantification of serum IgG specific to SARS-CoV-2 WT spike (AU/mL) through day 151 post-immunization. (**D**) Neutralizing antibody titers over 61 days post-vaccination, FRNT_50_. (**E**) Cross-reactive serum IgG levels against SARS-CoV-2 spike variants (WT, BA.1, and BA.5) at day 61 (AU/mL). Data represent individual mice with mean ± SEM; *P*-values determined by Welch’s *t*-test. (**F**) AUC of serum antibody (α'NP IgG2a) clearance in mock- (*N* = 3) and α'NP-injected SPF (*N* = 4) and dirty mice (*N* = 4), with standard deviation indicated. Each point represents *N* = 3–4 AUC values, aside from mock d59 (*N* = 2).

To investigate the humoral immune response to mRNA vaccines in the context of previous infection history, we vaccinated SPF and dirty mice with either Pfizer-BioNTech SARS-CoV-2 mRNA vaccine or mRNA encoding the receptor-binding domain (RBD) derived from the WA1 variant (Fig. 1A). This experiment was performed four times to ensure consistency across the unique microbial exposures from co-housing. A booster vaccination was performed 31 days (d31) post-prime. To determine if immune experience impacts antibody production after mRNA SARS-CoV-2 vaccination, serum IgG was evaluated for SARS-CoV-2 spike binding by enzyme-linked immunosorbent assay (ELISA). While SPF mice showed a robust IgG response to the prime that increased after the booster, dirty mice required the booster to reach a comparable IgG response (Fig. 1B). The antibody titer declined between 62 and 151 days post-vaccination at a slightly higher rate in the dirty compared to SPF mice, though the difference was not significant (Fig. 1C). Further, serum antibodies from vaccinated SPF mice better neutralized SARS-CoV-2 USA-WA1/2020 30 days post-booster, as determined by a fluorescence reduction neutralization test (Fig. 1D), and had improved the recognition of Omicron variants BA.1 and BA.5 (Fig. 1E). Additionally injecting mouse IgG antibody targeting the influenza A nucleoprotein (α′NP) into the tail veins demonstrated faster α′NP antibody reduction from the serum of dirty mice (Fig. 1F), suggesting that the clearance rate in dirty mice may act as a potential mechanism for reduced vaccine titers.

**Fig 2:**
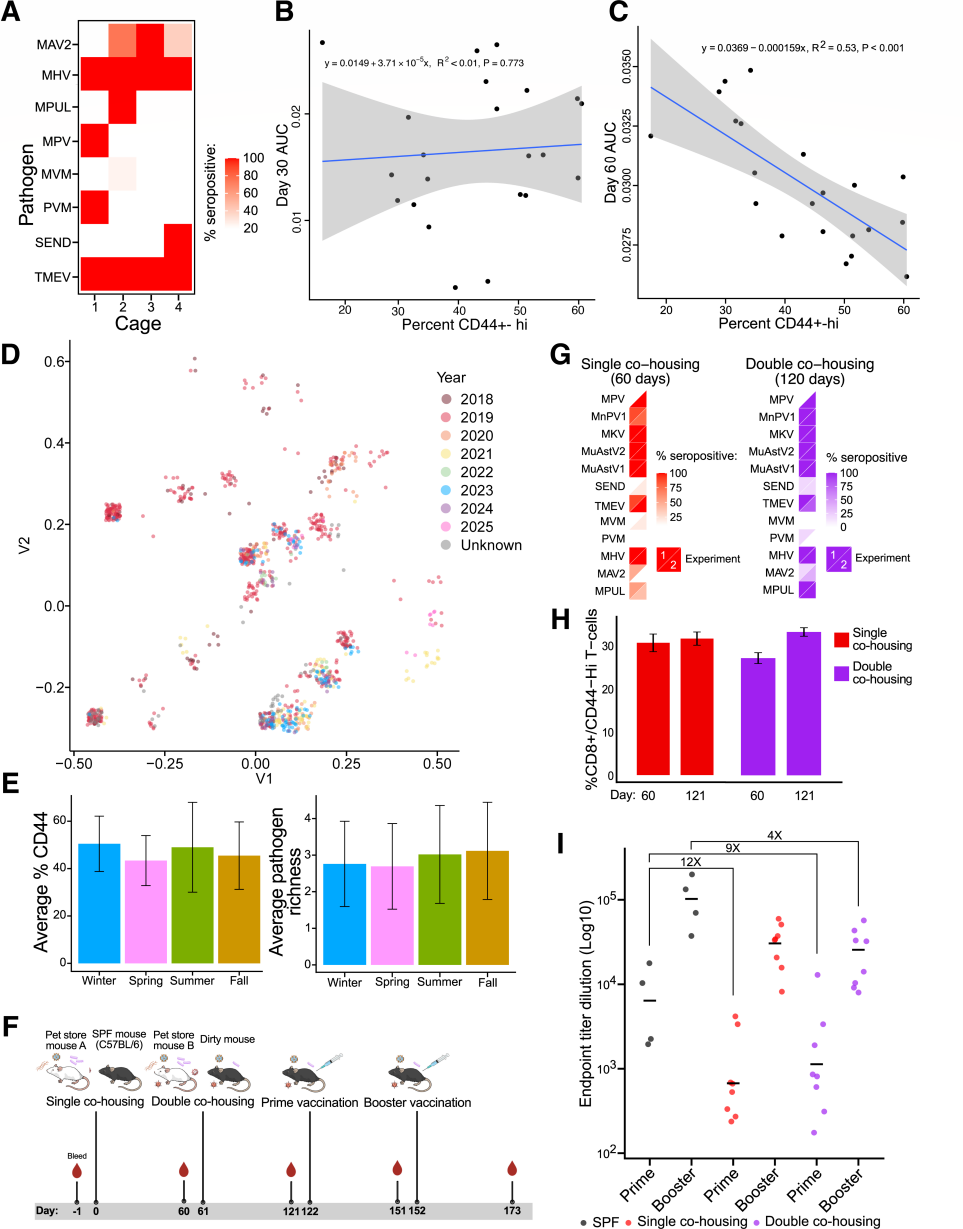
Stability of the dirty mouse model. (**A**) Pathogen seropositivity across co-housed cohorts (from Fig. 1B through E). (**B and C**) Correlation between CD8 +T cell activation (%CD44-Hi) and anti-S1 RBD IgG levels (AUC) 30 days post-prime (day 30, **B**) or 30 days post-boost (day 60, **C**), *N* = 21 mice. (**D**) Multidimensional scaling analysis of co-housed mouse serology (*N* = 1,014). The distance between the points represents the similarity of pathogen exposure history. Points are colored by experiment year. (**E**) Average %CD8a+/CD44+-Hi cells (left) and pathogen richness (right) across seasons (± SD). (**F**) Generation of double co-housed dirty mice. (**G**) Pathogen seroprevalence in single (60 days) and double (120 days) co-housed mice. Triangles represent independent experiments. (**H**) %CD8a+/CD44-Hi T cells in single and double co-housed mice at days 60 and 121 (mean ± SEM). (**I**) Anti-S1 RBD IgG log10 endpoint titers (OD_405_) 30 days post-prime/boost (*N* = 20) in SPF (black), single (red), and double (purple) co-housed mice. Data represent individual mice, bars represent mean, and brackets indicate fold-change relative to SPF.

We next assessed the pathogen burden in these animals and found diverse pathogens across four independent experiments (Fig. 2A). We measured the frequency of CD8a+/CD44 +T cells in the blood as a readout of immune system activation (2) to investigate whether activated T cell levels correlated with vaccine prime and boost response. Despite the broad range of vaccine responses in dirty mice after priming (Fig. 1B), we found no correlation between the percent of CD44-hi T cells and S1 RBD IgG (Fig. 2B). However, after the boost at day 61, we found a slight correlation between activated T cells and an attenuated response (*P* < 0.001, Fig. 2C), although the range of S1 RBD IgG was more limited than at day 30. We further evaluated the pathogen heterogeneity of the co-housing system across 8 years and all four seasons using data from 1,014 laboratory mice co-housed with pet store mice. We used multidimensional scaling to compare the similarity of pathogen exposure profiles of the mice, revealing heterogeneity in exposure (Fig. 2D). However, the overall T cell activation status was not different over time or season (Fig. 2E), suggesting the dirty mouse model is a highly robust model for immune system activation despite potential differing pathogen exposures. To investigate the effect of repeated microbial exposures on the immune response, we established a double co-housing model where SPF mice were co-housed with a pet store mouse for 60 days and then re-housed with a new pet store mouse for another 60 days (Fig. 2F). Despite re-exposure to many pathogens (Fig. 2G), double co-housing did not significantly increase the T cell activation compared to single co-housing (Fig. 2H), and there was no difference in post-booster antibody responses to the mRNA vaccine between the two models (Fig. 2I). However, the primary response was notably more heterogeneous in double co-housed mice, consistent with their more complex microbial exposure.

Despite the overwhelming success of SARS-CoV-2 mRNA vaccines at preventing hospitalization and death, their efficacy wanes over time (11) and requires a booster for optimal efficacy (12). Initial preclinical testing of SARS-CoV-2 mRNA vaccine candidates was performed in SPF mice and macaques raised in standard SPF conditions. Vaccination of these animals with mRNA vaccines resulted in robust neutralizing antibody titers (13, 14). Given that humans do not live in SPF conditions and are regularly confronted by pathogens, we investigated whether the humoral response in dirty mice vaccinated with SARS-CoV-2 mRNA vaccines better modeled the human response toward the vaccination. Dirty mice had lower spike IgG serum antibody titers after initial vaccination and experienced faster titer wane over time, while the resultant antibodies were not as effective at neutralizing WT SARS-CoV-2 or binding to Omicron variants BA.1 and BA.5, compared to SPF mice. These results are consistent with data from mice vaccinated with influenza virus vaccines (2) and demonstrate that co-housed mice are a valuable model for mRNA vaccines that have not been previously evaluated in dirty mice.

The mechanism underlying reduced mRNA vaccine response in dirty mice remains unclear. Rewilding mice results in greater B cell maturation and germinal center responses (15), but how B cell activation interferes with generating *de novo* antibodies remains unknown (2). We found that antibodies cleared faster in dirty compared to SPF mice, which may partially explain the enhanced durability by SPF vaccination. CD8 T cell activation status, while historically used to evaluate the “dirtiness” of cohoused mice (1, 2), was not a useful predictor of the antibody prime response and only moderately correlated with the response after a boost dose. This highlights the need to identify an additional correlate of vaccine response in dirty mice, with T follicular helper and B cells as priority targets.

Pathogen exposure history is heterogeneous over time in the pet store co-housing model but yields a robust T cell activation. We found no evidence of seasonal differences in T cell activation or pathogen exposure, which may affect other methods of microbial exposure that rely on wild mice or outdoor exposure. We found that pathogen number, combination, and T cell activation status were stable and comparable between 2018 and 2025, consistent with the stability over time of other dirty mouse platforms (16).

The basal immune signature of SPF mice closely matches that of neonatal humans (3). As individuals mature into adulthood, cumulative infections continue to shape the immune system, permanently altering memory T cell composition and expanding innate immune cell populations (1, 3). With the double co-housing model, we mimicked this scenario and found that pathogen recall did not affect the already established T cell activation in our model. The antibody response in the double co-housing model confirms our prior data and existing research (2, 3), where the exaggerated antibody titers typically produced in clean mice decreased as we increased the complexity of the immune system in the model.

The dirty mouse humoral response better models the human SARS-CoV-2 mRNA vaccination experience, given that humans require booster doses (12, 17), experience waning serum antibody titers (17–19), and reduced variant neutralization following vaccination (20, 21). This is consistent with previous results using other vaccine modalities, suggesting this is a general phenomenon that will translate to new vaccine approaches. SPF mice are often poor predictors of human immune responses, and more translationally representative dirty mice should be used for preclinical immunization studies due to their similarity to a more developed adult human immune system.

## Data Availability

The code for MDS analysis of pathogen exposure is located on github at github.com/langloislab/praena-shepherd-mcdonald-2026.

## SUPPLEMENTAL MATERIAL

Supplemental text mbio.00983-26-s0001.docx Supplemental materials and methods.

ASM does not own the copyrights to Supplemental Material that may be linked to, or accessed through, an article. The authors have granted ASM a non-exclusive, world-wide license to publish the Supplemental Material files. Please contact the corresponding author directly for reuse.
