# Supplementary material for: Dirty mice better recapitulate key features of mRNA vaccine immunogenicity observed in humans
Source: mBio. 2026 Jul 6;17(8):e00983-26. doi: 10.1128/mbio.00983-26 (PMC13463785; doi:10.1128/mbio.00983-26)
Supplement: Supplementary file 1 [file mbio.00983-26-s0001.docx]

**Supplemental Materials and Methods**

Co-housing and serological analysis of mice:

Age-matched female 8- to 10-week-old C57BL/6 (Jackson laboratory) mice were either housed in a specific pathogen-free (SPF) mouse facility or co-housed in a BSL-3 facility with mice acquired from a local pet store for 60 days, resulting in what is referred to as dirty mice. Thereafter, submandibular bleeding was performed on the mice to collect blood onto Opti-Spot strips (IDEXX BioAnalytics) for serological testing, whose panel tested for *Filobacterium rodentium,* *Encephalitozoon cuniculi* (ECUN), Ectromelia virus, Epizootic Diarrhea of Infant Mice/Mouse Rotavirus (EDIM), Lymphocytic Choriomeningitis Mammarenavirus (LCMV), *Mycoplasma pulmonis* (MPUL), Mouse Adenoviruses Type 1 (MAV1) and 2 (MAV2), Mouse Cytomegalovirus (MCMV), Mouse Hepatitis Virus (MHV), Mouse Parvovirus Type-1 (MPV), Minute Virus of Mice (MVM), Mouse Polyomavirus, Pneumonia Virus of Mice (PVM), Reovirus (REO), Theiler’s Murine Encephalomyelitis Virus (TMEV), Sendai Virus (SEND), and *Clostridium piliforme* (CPIL). In-house peptide ELISAs were developed to detect antibodies against viruses not typically included in commercial panels, specifically murine astrovirus 2 (MuAstV2), murine kobuvirus (MKV), and Minnesota rodent picornavirus 1 (MnPV1); these assays were performed as previously described (1, 2). For murine astrovirus 1 (MuAstV1), a commercial ELISA plate (XpressBio) pre-coated with recombinant capsid protein was utilized. Blood was additionally collected for PBMC (peripheral blood mononuclear cell) isolation and flow analysis for markers of immune cell identity and activation. CD8a+/CD44+-Hi cells were first gated to identify single cells, then to identify CD45+ immune cells (anti-CD45 Brilliant Violet 711 antibody, clone30F-11, BioLegend), then to identify CD8a+ cells (anti-CD8a PE/Cyanine5 antibody, clone 53-6.7, BioLegend), and finally to identify KLRG1+/CD44+-Hi cells (anti-CD44 Brilliant Violet 785 antibody, clone IM7, BioLegend; anti-KLRG1 PE/Cyanine7 antibody, clone 2F2, BioLegend). Cells were analyzed using a BD LSRFortessa™ flow cytometer (BD Biosciences), with a minimum of 100,000 live events acquired per sample. For the double co-housing model, a subset of the dirty mice from the initial 60-day exposure was subjected to a second 60-day co-housing period (double Co-Ho) prior to immunization. The Institutional Animal Care and Use Committee (IACUC) of the University of Minnesota approved all animal experimentation performed in this work.

Vaccination/serum collection:

Mice were vaccinated intramuscularly in the rear leg with either 0.25 µg Pfizer-BioNTech COVID-19 vaccine (PAA165969, Lot EN6025) or with 0.5 µg of mRNA encoding the SARS-CoV-2 receptor-binding domain (RBD) derived from the WA1 variant (2020 SARS-CoV-2 A.1 strain mRNA vaccine), formulated in lipid nanoparticles, and delivered in PBS for a total volume of 50 µl. The vaccine design was in accordance with World Health Organization (WHO) recommendations for variant-based COVID-19 immunogens. Vaccination occurred 61-113 days post-co-housing in 24-week-old female mice. Mice were primed (day 0) and provided an identical booster vaccination after 31 days. For the double co-housing, mice were primed on day 120 post-co-housing, corresponding to 60 days after completion of the first co-housing protocol, and received a homologous booster immunization 31 days later. Submandibular bleedings were performed on the days immediately prior to vaccination (d0), 30 days post-prime prior to the booster dose (d30), 30 days post-boost (d61-62), and monthly after the booster vaccination (d91, d122, and d151) until the end of the experiment. Serum was isolated from collected blood following one hour of coagulation at room temperature, followed by 15 minutes' centrifugation at 3,000 x G, 4ºC. Serum was stored at -80ºC until experimental use.

Vaccine-specific antibody detection

Vaccine-specific antibodies were detected with either ELISA or the 10-plex Meso Scale Discovery-Electrochemiluminescence immunoassay (MSD-ECLIA), as stated in figure legends. For ELISA, 96-well plates (Corning Inc.) were coated with 3 µg/mL of recombinant SARS-CoV-2 S1 RBD protein (3) diluted in phosphate-buffered saline (PBS; Bio-Rad Laboratories, Inc.) overnight at 4ºC. The following day, plates were washed three times with PBS containing 0.5% Tween-20 (PBS-T; Hoefer, Inc.), then blocked with 1% bovine serum albumin (BSA; MP Biomedicals) in PBS for 1 hour at room temperature to prevent nonspecific binding. After blocking, plates were washed again three times with PBS-T. Mouse serum was diluted 1:50 in dilution buffer (PBS containing 0.05% Tween-20 and 0.2% BSA), then serially diluted 1:4 in dilution buffer, added to the plates, incubated for 1 hour at 37ºC, and subsequently washed three times with PBS-T. Bound serum antibodies were detected by incubating the plate for 1 hour at 37ºC with horseradish peroxidase (HRP)-conjugated anti-mouse IgG antibodies (Southern Biotech) diluted 1:500 in blocking buffer, and the plate was subsequently washed four times in PBS-T. Plates were then incubated with ABTS peroxidase substrate (SeraCare) for 45 minutes at room temperature in the dark, and reaction was stopped with 2% sodium dodecyl sulfate (SDS; Fisher Scientific) in PBS. For visualization, optical density at 405 nm (OD405) was measured using a Synergy H1 microplate reader (BioTek). OD405 values were plotted against the dilution factor, and the area under the curve (AUC) was calculated using GraphPad Prism 10.

For MSD-ECLIA (Rockville, MD), SARS-Cov2 spike IgG was quantified using plates coated with WA.1, BA.1, or BA.5 spike proteins (4). Briefly, plates were blocked with 5% BSA for 60 minutes prior to the addition of samples diluted at 1:5000 and allowed to incubate with shaking for two hours. Then, plates were washed and labeled with Sulfo-tagged anti-IgG antibody. Plates were washed to remove unbound detection antibodies and binding was detected via electrochemiluminescence on an MSD Sector instrument after addition of ECL substrate. Raw data were analyzed using Discovery Workbench and GraphPad Prism software v10.1.2. The antibody concentrations were calculated relative to antibody standard controls and expressed in arbitrary units (AU) per mL for IgG.

Cells and Viruses.

VeroE6-TMPRSS2 cells were generated and cultured as previously described (5). All SARS-CoV-2 viruses were plaque purified and propagated once in VeroE6-TMPRSS2 cells to generate working stocks. Viruses were deep sequenced and confirmed as previously described (6).

Focus Reduction Neutralization Test (FRNT)

FRNT assays were performed as previously described (5-7). Briefly, serum samples were 3-fold diluted in 8 serial dilutions using DMEM with an initial dilution of 1:10. Serially diluted samples were mixed with an equal volume of the desired SARS-CoV-2 variant (100-200 foci per well). The virus-serum mixtures were incubated at 37°C for 1 hour in a round bottom 96-well culture plate. After 1 hour incubation, the virus-serum mixture was added to VeroE6-TMPRSS2 cells and incubated at 37°C for an additional hour. Post-incubation, the mixture was removed from cells and 100μl of prewarmed 0.85% methylcellulose overlay was added to each well. Plates were incubated at 37°C for 18 to 40 hours (depending on the SARS-CoV-2 strain). After the appropriate incubation time, the methylcellulose overlay was removed, and cells were washed with PBS and fixed with 2% paraformaldehyde for 30 minutes. Following fixation, cells were washed twice with PBS and permeabilized using permeabilization buffer for at least 20 minutes. After permeabilization, cells were incubated with an anti-SARS-CoV-2 spike primary antibody directly conjugated to Alexa Fluor-647 (CR3022-AF647) overnight at 4°C. Cells were then washed twice with 1X PBS and imaged on an ELISPOT reader (CTL Analyzer).

Seasonality analysis

Serology testing results from this study were combined with historical serology panel testing data from dirty mice. Historical data included C57BL/6 female mice that were co-housed with pet store mice, Balb/c female mice that were co-housed with pet store mice, and C57BL/6 male mice that were exposed to pet store mice fomites (male mice cannot be co-housed with pet store mice due to ethical concerns). Historical data was collected from 2018-2024 and includes previously published serology data (8). In total, 1014 data points were included in the analysis. Serology panel data were combined and limited to the pathogens shared in all panel assays and included CPIL, ECUN, EDIM, TMEV, LCMV, MAV, MCMV, MHV, MPUL, MPV, MVM, POLY, PVM, REO, and SEND. The IDEXX panel used for this work included separate tests for MAV1 and MAV2, and these tests were collapsed to a single MAV result (if mice were positive for either MAV1 or MAV2, they were considered MAV-positive). Historical panels tested for several serotypes of MPV in separate, and these data were similarly collapsed to a single MPV result to combine with the panel data used in this study (if mice were positive for any of the MPV targets, they were considered MPV-positive). Serology panel data (binary data of either seropositive or seronegative) were used for multidimensional scaling analysis using the stats package in R (9). The percentage of CD8a+/CD44+-Hi T cells were compared across the 1014 mice to identify differences in seasonality of T cell activation status. The number of seropositive pathogens per mouse was also compared across seasons.

Statistical analyses

To compare rates of vaccine-specific antibody decline (Fig. 1C), a linear regression was fit to SARS-CoV-2 WT spike IgG levels (AU/ml) for the dirty and SPF mice for data collected at 62-, 91-, 122-, and 151-days post-priming. The slopes were compared using Welch’s T test. The percentage of CD8a+/CD44+-Hi T cells was correlated with antibody levels (measured by AUC) on day 30 (30 days post-prime) and day 60 (30 days post-boost) using Pearson correlation. Antibody neutralization was quantified by counting the number of foci for each sample using the Viridot program (10). The neutralization titers were calculated as follows: 1 – (ratio of the mean number of foci in the presence of sera and foci at the highest dilution of the respective sera sample). Each sample was tested in duplicate. The FRNT-50 titers were interpolated using a 4-parameter nonlinear regression in GraphPad Prism 10.1.2. Samples that do not neutralize at the limit of detection at 50% are plotted at 20 and used for geometric mean and fold-change calculations.

Antibody clearance

Mice were tail vein-injected either with 200 µg influenza A nucleoprotein-targeting (α'NP) antibody (clone H16-L10-4R5 (HB-65); BioXCell) brought to 100 µl total with InVivo Pure pH 7.0 dilution buffer (BioXCell), or with 100 µl dilution buffer alone for the mock condition. In total, three mock SPF mice, four α'NP-injected SPF mice, and four α'NP-injected dirty mice were used. Submandibular bleedings of mice were performed one week prior to injection (d-7), the day following injection (d1), and 2, 4, 6, 8, 12, and 17 weeks after injection (d14, d29, d43, d59, d90, d120). To assess serum α'NP antibody levels, ELISA assays were performed as previously described on plates coated with 1 µg/ml His-tagged Influenza A H1N1 (A/Puerto Rico/8/34/Mount Sinai) Nucleoprotein (I116M; Sino Biological, Inc.) diluted in PBS. Serum was diluted 1:100 in dilution buffer prior to serial 1:4 dilutions, and Goat Anti-Mouse IgG2a Human ads-HRP secondary antibody (SouthernBiotech) was used as the secondary antibody for detection. AUC and standard deviation per timepoint were calculated and plotted using GraphPad Prism 10.

Figure preparation

The following NIH BioArt (11) images were used or modified to generate Figure 1A and Figure 2E: BIOART-000042, BIOART-000279, BIOART-000464, BIOART-000496, BIOART-000505, BIOART-000646, and BIOART-000652.

**Supplemental References**

1.	Sanders AE, Arnesen H, Shepherd FK, Putri DS, Fiege JK, Pierson MJ, Roach SN, Carlsen H, Masopust D, Boysen P, Langlois RA. 2024. Comparison of mouse models of microbial experience reveals differences in microbial diversity and response to vaccination. mSphere 9:e0065423.

2.	Putri DS, Shepherd FK, Sanders AE, Roach SN, Jay S, Pierson MJ, Wieking G, Anderson JL, Meyerholz DK, Schacker TW, Langlois RA. 2025. Naturally transmitted mouse viruses highlight the heterogeneity of virus transmission dynamics in the dirty mouse model. J Virol:e0018725.

3.	Pape KA, Dileepan T, Kabage AJ, Kozysa D, Batres R, Evert C, Matson M, Lopez S, Krueger PD, Graiziger C, Vaughn BP, Shmidt E, Rhein J, Schacker TW, Khoruts A, Jenkins MK. 2021. High-affinity memory B cells induced by SARS-CoV-2 infection produce more plasmablasts and atypical memory B cells than those primed by mRNA vaccines. Cell Rep 37:109823.

4.	Pegu A, O'Connell SE, Schmidt SD, O'Dell S, Talana CA, Lai L, Albert J, Anderson E, Bennett H, Corbett KS, Flach B, Jackson L, Leav B, Ledgerwood JE, Luke CJ, Makowski M, Nason MC, Roberts PC, Roederer M, ..., Group§ m-S. 2021. Durability of mRNA-1273 vaccine-induced antibodies against SARS-CoV-2 variants. Science 373:1372-1377.

5.	Edara VV, Norwood C, Floyd K, Lai L, Davis-Gardner ME, Hudson WH, Mantus G, Nyhoff LE, Adelman MW, Fineman R, Patel S, Byram R, Gomes DN, Michael G, Abdullahi H, Beydoun N, Panganiban B, McNair N, Hellmeister K, ..., Suthar MS. 2021. Infection- and vaccine-induced antibody binding and neutralization of the B.1.351 SARS-CoV-2 variant. Cell Host Microbe 29:516-521 e3.

6.	Edara VV, Pinsky BA, Suthar MS, Lai L, Davis-Gardner ME, Floyd K, Flowers MW, Wrammert J, Hussaini L, Ciric CR, Bechnak S, Stephens K, Graham BS, Bayat Mokhtari E, Mudvari P, Boritz E, Creanga A, Pegu A, Derrien-Colemyn A, ..., Fabrizio TP. 2021. Infection and Vaccine-Induced Neutralizing-Antibody Responses to the SARS-CoV-2 B.1.617 Variants. N Engl J Med 385:664-666.

7.	Vanderheiden A, Edara VV, Floyd K, Kauffman RC, Mantus G, Anderson E, Rouphael N, Edupuganti S, Shi PY, Menachery VD, Wrammert J, Suthar MS. 2020. Development of a Rapid Focus Reduction Neutralization Test Assay for Measuring SARS-CoV-2 Neutralizing Antibodies. Curr Protoc Immunol 131:e116.

8.	Fiege JK, Block KE, Pierson MJ, Nanda H, Shepherd FK, Mickelson CK, Stolley JM, Matchett WE, Wijeyesinghe S, Meyerholz DK, Vezys V, Shen SS, Hamilton SE, Masopust D, Langlois RA. 2021. Mice with diverse microbial exposure histories as a model for preclinical vaccine testing. Cell Host Microbe 29:1815-1827.e6.

9.	Team RC. 2025. R: A Language and Environment for Statistical Computing, R Foundation for Statistical Computing, <https://www.R-project.org/>.

10.	Katzelnick LC, Coello Escoto A, McElvany BD, Chavez C, Salje H, Luo W, Rodriguez-Barraquer I, Jarman R, Durbin AP, Diehl SA, Smith DJ, Whitehead SS, Cummings DAT. 2018. Viridot: An automated virus plaque (immunofocus) counter for the measurement of serological neutralizing responses with application to dengue virus. PLoS Negl Trop Dis 12:e0006862.

11.	NAIAD Visual and Medical Arts. 2026. NAIAD BioArt Source.
